# Human Mesenchymal Stem Cells: The Present Alternative for High-Incidence Diseases, Even SARS-Cov-2

**DOI:** 10.1155/2020/8892189

**Published:** 2020-12-18

**Authors:** Karen J. Juárez-Navarro, Eduardo Padilla-Camberos, Néstor Fabián Díaz, Ariel Miranda-Altamirano, N. Emmanuel Díaz-Martínez

**Affiliations:** ^1^Biotecnología Médica y Farmacéutica Centro de Investigación y Asistencia en Tecnología y Diseño del Estado de Jalisco, Guadalajara, Mexico; ^2^Departamento de Fisiología y Desarrollo Celular, Instituto Nacional de Perinatología Isidro Espinosa de los Reyes, Mexico City, Mexico; ^3^Departamento de Atención a Niños con Quemaduras, Hospital Civil de Guadalajara, Mexico

## Abstract

Mesenchymal stem cells (MSCs), defined as plastic adherent cells with multipotent differentiation capacity in vitro, are an emerging and valuable tool to treat a plethora of diseases due to their therapeutic mechanisms such as their paracrine activity, mitochondrial and organelle transfer, and transfer of therapeutic molecules via exosomes. Nowadays, there are more than a thousand registered clinical trials related to MSC application around the world, highlighting MSC role on difficult-to-treat high-incidence diseases such as the current COVID-19, HIV infections, and autoimmune and metabolic diseases. Here, we summarize a general overview of MSCs and their therapeutic mechanisms; also, we discuss some of the novel clinical trial protocols and their results as well as a comparison between the number of registries, countries, and search portals.

## 1. Introduction

Stem cells are undifferentiated cells with remarkable qualities such as unlimited self-renewal capacity and differentiation potential [1]. Nowadays, stem cells are an emerging tool for cell-based therapy in a variety of diseases; however, ethical controversy and safety assessment of embryonic stem cells (ESCs) remain unsolved; due to this, the exploration of nonembryonic stem cells as a therapeutic resource has increased significantly over the last years. Mesenchymal stem cells (MSCs) are a type of stem cells characterized by their multipotent differentiation potential into chondrogenic, adipogenic, and osteogenic cell lineages as well as their high proliferative rates that allow researchers to reach the needed amount of cells promptly [2]. MSCs are mainly characterized by three minimum criteria that are focused on their phenotype and potency [3]. Also, MSCs are well known for their therapeutic effects involving their differentiation potential and mostly their paracrine activity and immunomodulatory capacity. Since 2004, the number of registered clinical trials employing MSCs has been increasing significantly to date, and despite being an emerging tool, the concentration of registries remains in the United States of America, China, and Europe [4].

The rationale of this review is to summarize and provide helpful information about mesenchymal stem cell therapeutic mechanisms and their biological effects, also, to provide recent clinical trial results and findings related to MSC application where high-incidence diseases are highlighted, and finally to summarize and compare trends of MSC clinical registries around the world. All these contribute to a better understanding of MSCs and to improve future clinical approaches on MSC application.

## 2. Mesenchymal Stem Cells: General Overview

Around the decades 1960s and 1970s, Friedenstein discovered mesenchymal stem cells; he stated that the bone marrow (BM) is a reservoir of multipotent stem cells by obtaining and implanting mouse femoral BM fragments under adult mouse renal capsule using diffusion chambers; results showed developing osteogenesis by the third day, and by the eighth day, the osteogenic foci had increased in size; furthermore, Friedenstein also isolated and assessed the proliferation capacity of guinea pig BM cells with fibroblast-like morphology by determining the concentration of colony-forming cells, now known as colony-forming unit fibroblast (CFU-F) concluding that there were stem cells with the ability to develop osteogenic precursors in BM and that differentiation might have been a result of the interaction within a community of cells [5–8]. Also, Friedenstein reported that when isolating fibroblast-like cells from femoral BM cells and spleen of guinea pig and rabbits and culturing these cells in vitro for several passages, they retained the capacity to form stromal tissue and when retransplanted under kidney capsule or as a cell suspension in a diffusion chamber bone formation was developed; on the other hand, retransplanted fibroblast-like cells isolated from the spleen gave rise to reticular tissue [9–11]. These were the first glimpses of MSCs; however, Friedenstein named them “osteogenic stem cells”; it was not until the 1990s that the term mesenchymal stem cell was proposed [9].

Nowadays, according to the International Society for Cellular Therapy (ISCT), the term mesenchymal stem cell is designated to “the plastic adherent cells isolated from BM and other tissues with multipotent differentiation capacity *in vitro*” and must fulfill three minimum criteria that are as follows. First of all, cells must exhibit plastic adherence; second, they should express the “CD105, CD73, and CD90 cell surface markers and show a lack of expression of the surface molecules CD45, CD34, CD14 or CD11b, CD79alpha or CD19, and HLA-DR,” and third, they must differentiate into adipocytes, chondroblasts, and osteoblasts [3, 12]. MSCs, as the other stem cell types, can proliferate extensively and differentiate into several cell line types including osteocytes, adipocytes, chondrocytes, myocytes, cardiomyocytes, hepatocytes, and neurons, depending on their source [13, 14].

**Figure 1:**
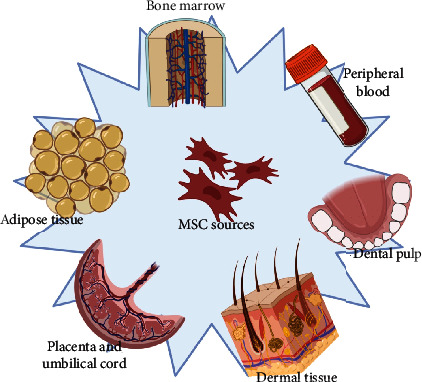
Sources of mesenchymal stem cells. Created with http://Biorender.com.

Sources to obtain MSCs are various as shown in Figure 1: BM, adipose tissue (AT), dental pulp, peripheral blood, and birth-derived tissues as placenta, umbilical cord blood and tissue, Wharton's jelly, and amniotic fluid, each one having specific differentiation potentials as shown in Table 1 [13, 15].

Other sources of MSCs that are being explored are ESC and induced pluripotent stem cell- (iPSC-) derived MSC, ESC-MSC, and iPSC-MSC, respectively. These have shown to overcome the aging-related issues and limited proliferation rate of adult MSCs; besides, iPSC-MSC show rejuvenation signs [16–18].

## 3. Mesenchymal Stem Cell Therapeutic Mechanisms

It is well known that the therapeutic effects of MSCs are owed to their immunomodulatory capacity derived from their paracrine function and their differentiation potential. The paracrine function of MSCs was proven for the first time by Gnecchi in 2006; a previous work had demonstrated that intramyocardial injection of BM-MSC restored cardiac function after two weeks post infarction; later, Gnecchi analyzed the therapeutic effects of the injection of conditioned medium obtained from genetically engineered MSC with the survival gene Akt1 (Akt-MSC) on rat cardiomyocytes exposed to hypoxic conditions; results proved that the therapeutic activity of Akt1-MSC was owed to MSC secretome [37, 38]. The secretome referred to as proteins or other types of factors secreted by cells can include growth factors (GFs), cytokines, chemokines, and extracellular vehicles (EVs) as shown in Table 2 [39, 40]. The immunosuppressive capacity of MSC secretome makes them an ideal tool to treat diseases associated with critical inflammation; however, it should be noted that secretome products may vary between different media conditions, cells, and sources.

An example of MSC therapeutic potential via paracrine activity is the restoration of corneal transparency after injection of BM-MSCs on a mouse model of injured corneas. Hepatocyte growth factor (HGF) secreted by MSC contributes to the restoration of transparency with or without the administration of MSCs [41]. Moreover, previous researches have shown that the paracrine effect of MSCs has a predominant role unlike cell differentiation (with a low differentiation efficiency); for instance, in cardiovascular diseases, MSC paracrine effects lead to angiogenesis and therefore contribute to restore heart function, and MSC secretome itself can be responsible for enhancing muscle regeneration [42–44].

**Figure 2:**
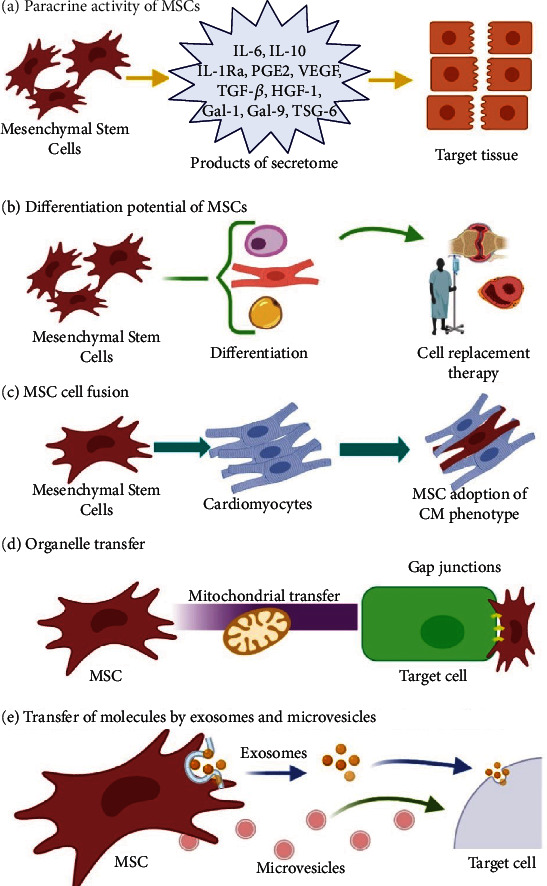
Mesenchymal stem cell therapeutic mechanisms: (a) the paracrine activity of MSCs, (b) differentiation potential of MSCs, (c) MSC cell fusion, (d) organelle transfer, and (e) transfer of molecules by exosomes and microvesicles. Created with http://Biorender.com.

In addition to the paracrine activity of MSCs, there are other therapeutic mechanisms shown in Figure 2, as the differentiation potential used in cell replacement therapy and regenerative medicine (Figure 2(b)). This kind of therapy is mainly focused on the reconstruction or regeneration of damaged tissues, principally the ones related to the nervous system, myocardium, liver, cornea, trachea, and skin. Corneal reconstruction has been possible in rabbits by MSC transplantation [67]. MSCs might also spontaneously fuse to somatic cells enhancing their differentiation and inducing tissue-specific function (Figure 2(c)); this therapeutic mechanism is applied to the repair of damaged tissues, and this has been proved in an *in vivo* mouse model of damaged myocardium where MSCs fused to cardiomyocytes (CM) and adopted a CM-like phenotype [68]. Organelle transfer by MSCs is also considered a therapeutic mechanism (Figure 2(d)). Mitochondrial transfer is noted due to its role in rescuing mitochondrial respiration abnormalities, signaling, proliferative regulation, and defect repairs [68, 69]. Mitochondria can be transferred from MSCs in unidirectional and bidirectional ways to injured cells by several mechanisms, mostly by the formation of tunnel tubes (TNTs), extracellular vesicles, cellular fusion, and GAP junctions [68, 70–73].

Molecular mechanisms of mitochondrial transfer have not been fully described yet; however, some molecules and processes associated with this transfer are known. For instance, the gap junction protein Connexin 43 (Cx43) plays an important role in TNT formation for mitochondrial transfer, and overexpression of this enhances TNT formation [74]. Furthermore, Miro-1 is also considered an important contributor for mitochondrial transfer; it is believed that migration of mitochondria through TNTs takes place via microtubes containing this protein [75].

Another relevant factor for mitochondrial transfer is proinflammatory cytokine secretion of damaged cells such as TNF*α*, IL-6, IL-8, and IL-1 and activation of the NF-*κ*B signaling pathway; these are highly present on the damaged environment and trigger mitochondrial transfer from MSCs. It has been observed that inflammation enhances and improves mitochondrial transfer and that somehow cell environment and cell to cell contact is important to enhance therapeutic mechanisms of MSCs [76, 77].

Experiments have revealed efficient mitochondrial transfer from MSCs to stressed corneal epithelial cells (CECs); also, mitochondrial transfer from iPSC-MSC to airway smooth muscle cells (ASMCs) with mitochondrial dysfunction has shown inflammation reduction on in vitro and in vivo mouse models [78, 79]. The mitochondrial transfer has the potential to prevent apoptosis of stressed or damaged cells which shows promise as a therapy for conditions associated with mitochondrial dysfunction as neural degeneration, corneal damage, and failures on cardiovascular, respiratory, and renal systems among others [79–82]. In addition, recent findings state that mitochondrial transfer from MSC to T cells modulates T cell function and transcriptomic profile resulting in a guided proliferation and migration, which leads to inflammation decrease and a lower number of T cells [83].

MSCs might also transfer therapeutic molecules via exosome and microvesicle release (Figure 2(e)). Exosomes transfer miRNAs and proteins related to immunoregulation and epigenetic regulation. Recently, it has been reported that human MSC-derived exosomes alleviate liver fibrosis in a rat model after *in vivo* administration [84].

## 4. Current Mesenchymal Stem Cell Research for Clinical Application

Owing to their therapeutic effects, mesenchymal stem cells and their secretome are being studied in a plethora of diseases that involve skin pathologies, cardiovascular diseases, neurologic pathologies, metabolic disorders, spinal cord injury, and autoimmune diseases.

### 4.1. Skin Pathologies

Recently, it was demonstrated that MSC conditioned media ameliorate psoriasis, an autoimmune, chronic inflammatory disease [85]. In a case study of a male patient with severe psoriasis, a topical treatment of adipose tissue-MSC conditioned media was performed for a month and complete regression was recorded during this time; similar to this, another case study wherein the treatment was performed with allogenic gingiva-MSC bolus injection in a patient with severe plaque psoriasis showed fading of psoriatic lesions six weeks after treatment, no abnormalities were reported, and the patient has been psoriasis-free for 3 years [86, 87]. Based on the pathogenesis of psoriasis and the fact that it is caused mainly by the increased release of proinflammatory cytokines, it could be inferred that the main mechanism of MSCs under these diseases is their paracrine activity, which, in this case, would be responsible for the decrease of inflammation associated to psoriasis. Nowadays, there are eight registered clinical trials at Clinicaltrials.gov that contemplate intravenous injection of mesenchymal stem cells; sources for the trials are mainly based on UC-MSCs while the rest is based on adipose-tissue MSCs (AD-MSCs) [88].

### 4.2. Autoimmune Diseases

Systemic lupus erythematosus (SLE), a syndrome involving alterations on the immune system [89], has also been treated with allogeneic BM-MSCs derived from healthy patients with no autoimmune diseases; in this study, 81 patients with severe and drug-refractory lupus erythematosus (SLE) received infusions of MSCs; results showed disease amelioration, and after a 5-year follow-up, 37 patients were in clinical remission [90]. Moreover, Barbado and colleagues [91] reported compassionate treatment with allogeneic MSCs from healthy patients for three chronic SLE patients; results were encouraging as one patient achieved a partial remission while the rest complete clinical remission. As in psoriasis trials, improvements in human SLE condition after MSC infusions are due to MSC immunomodulatory capacity; in lupus mouse models, UC-MSCs have shown to promote lymphocyte apoptosis and a decrease in T helper cells; this would explain the reduction of inflammation in SLE patients after treatment (Huang, 2020). Around 14 registered clinical trials involving MSCs to treat SLE can be found at the U.S. National Library of Medicine (at Clinicaltrials.gov); however, trials have not posted results yet.

Moreover, the application of UC-MSCs on 13 HIV-1 difficult-to-treat patients was also explored; seven patients received UC-MSC transfusion; results showed increased CD4 T cell counts and restoration of HIV-1-specific IFN-*γ* and IL-2 production [92]. However, there are only two registered clinical trials (at Clinicaltrials.gov) of MSC treatment to fight HIV with no results posted. To date, there is no fine information that defines the exact therapeutic mechanism of MSCs on HIV infections; nevertheless, it is possible that MSC transfusion restores CD4 T cell counts and reconstitutes the immune system, but it would be important that future experiments investigate whether this conjecture is true and to further explain molecular mechanisms involved in this.

Furthermore, diabetes mellitus (DM) type I condition, an autoimmune disorder that destroys *β* cells, also improves in experimental mouse models after intrapancreatic transplantation of mesenchymal stem cells; this is owed to the differentiation of MSC towards pancreatic cells and the possible enhancing and activation of pancreatic stem cells, all together would propitiate a better microenvironment. It is important to highlight that the MSC environment and cell-cell contact are important; for instance, direct administration of MSC on pancreatic injury provides better outcomes; additionally, after engraftment of MSCs, these are “guided” to the injured site by pancreas-secreted chemokines [93, 94]. There are currently three clinical trials running; one treatment is based on MSC exosome therapy on *β* cell mass, whereas the rest employs MSC intravenous infusion. Unlike intravenous MSC treatment, exosome therapy has shown better outcomes and improvements on type 1 DM owing to the genetic and protein contents that can trigger repair mechanisms [95].

### 4.3. Cardiac Diseases

MSCs are well known to have potential benefits on heart diseases, and several clinical studies have been developed to assess the safety and efficacy of MSC administration. For instance, it was reported that in a randomized study, patients with nonischemic cardiomyopathy received ischemia-tolerant MSCs (itMSCs); itMSC therapy showed improvements in patient health status, primarily owed to the immunomodulatory and anti-inflammatory activity of MSCs [96]. Also, the safety of transendocardial stem cell injection of autologous MSCs on ischemic cardiomyopathy patients has been tested in a study including 65 patients. There was an improvement on the Minnesota Living with Heart Failure score, scar reduction, and left ventricular (LV) function on the MSC treatment; in this case, MSCs might have promoted either cardiomyocyte regeneration, neovascularization, or endogenous stem cell proliferation as the observations from animal models, all this due to MSC paracrine function which has proangiogenic, antiapoptotic, and recruiting of stem cell effects [43, 97].

### 4.4. Musculoskeletal Diseases (MSDs)

Considered as one of the leading contributors to disability worldwide, musculoskeletal disorders include osteoarthritis, rheumatoid arthritis, low back pain, osteoporosis, sarcopenia, and myofascial pain syndrome [98]. The safety of MSC treatment in humans with osteoarthritis, an MSD and a chronic inflammatory joint disease of high prevalence [99], has been assessed in a 12-patient study; all of the patients had chronic knee pain. Treatment was performed by intra-articular injection of autologous expanded BM-MSCs; 91% of the patients showed improvement of cartilage quality [100]. Also, allogenic transplant of BM-MSCs has been studied as well in a clinical trial where the test group was treated with an intra-articular injection of allogeneic BM-MSCs; this study also showed cartilage quality increase [101]. Favorable outcomes are owed to the chondrogenic differentiation potential of MSCs; also, in vitro assays have shown that the microenvironment of the injured site induces MSCs to express key genes in the development of cartilage and synthesis of collagen II, all together promoting cartilage regeneration [102].

### 4.5. Nervous System Diseases

MSC application in spinal cord injury (SCI) and its repercussions has also been tested; Jiang and colleagues reported encouraging results after autologous expanded BM-MSC transplantation into 20 patients; results showed significant improvements in sensory, motor, and autonomic nerve function [103]. In another study, the subarachnoid administration of autologous MSCs was tested in ten SCI patients at months 1, 4, 7, and 10 of the study. Results showed that 100% of the patients improved their sensitivity and motor function, and neuropathic pain disappeared on half of the patients suffering from it, whereas the rest showed decreased pain [104]. Despite the fact that the exact mechanism of MSC on SCI is not fully understood yet, these positive outcomes are probably owed to MSC secretion of neurotrophic growth factors and the synthesis of myelin into the demyelinated spinal cord [103, 105].

Moreover, the effect of MSCs on diabetic neuropathy has also been assessed. Treatment was performed with intravenous injection of expanded autologous BM-MSCs in ten patients. Levels of b-FGF and v-EGF were measured to determine the effect of MSCs in nerve regeneration; results showed a significant increase after 90 days. Also, nerve conduction velocities of nerves increased. No adverse effects were observed [106].

### 4.6. Graft vs. Host Diseases

Graft-versus-host disease (GVHD) is a complication of allogeneic hematopoietic stem cell transplantation (HSCT) that occurs due to the presence of donor's immunocompetent cells; this condition is considered a major obstacle of HSCT avoiding successful treatment of hematological diseases; that is why novel approaches such as MSCs to overcome this challenge must be considered [107, 108].

Recently, a successful treatment of steroid-refractory gastrointestinal acute graft-versus-host disease (AGVHD) with MSCs was reported. After HSCT, the patient developed severe diarrhea, high fever, and erythema despite steroid therapy. Intravenous infusions of MSCs were administered 12 times in total. After MSC therapy, diarrhea diminished, and restoration of gastrointestinal damage was observed [109]. In another clinical trial where 25 steroid-AGVHD patients were enrolled, results showed 24% and 36% complete response and partial response, respectively [110]. Promising outcomes are primarily owed to the immunomodulatory capacity of MSCs; for instance, MSCs reduce the number of T-helper 17 cells, which produce critical proinflammatory cytokines on GVHDs [109]. There are currently 43 registered clinical trials to treat GVHD with MSC on the search portal Clinicaltrials.gov; however, just one trial has uploaded its results, 30% are completed, and the remaining are still recruiting or in an unknown status.

### 4.7. Cancer

Currently, there are 59 registered trials to treat cancer and other neoplasms with MSCs (at Clinicaltrials.gov); however, experimental assays have shown big discrepancies between the possible outcomes of MSCs on cancer treatment; both antitumor and protumorigenic activities have been shown. For instance, *in vitro* coculture of human bone marrow MSCs (hBMSCs) on human glioma cells has shown cell cycle arrest and apoptosis of glioma cells [111]. On the other hand, experimental approaches have also demonstrated the risk of cancer progression when using MSCs; hBMSC conditioned medium (hBMSC-CM) was applied to hepatocellular carcinoma cells, and increased cell invasion was shown [48]. Thus, the safety of MSC application on cancer treatment research remains unclear, and antitumor and protumorigenic activity may depend on the cancer type, MSC source, dose, timing of MSC, and pathological and genetic conditions of each patient. Zero out of the current 59 registered clinical trials has posted results.

## 5. Mesenchymal Stem Cells' Current Role in the COVID-19 Pandemic

SARS-Cov2 virus declared as a global emergency by the World Health Organization was detected for the first time in December 2019 in China, and it is the cause of an acute respiratory syndrome that has already taken 1,211,986 lives and 47,362,304 cases were confirmed around the world [112].

According to the World Health Organization, there is no specific and efficient treatment to combat this viral infection, and patients are mostly treated with drugs that mitigate symptoms. Furthermore, treatment varies depending on the patient's condition; for mild COVID-19 patients, symptomatic treatment is given, while patients with severe COVID-19 require oxygen therapy and treatment to counteract coinfections and symptoms of acute respiratory distress syndrome (ARDS) [113]. Moreover, despite promising results of current experimental treatments to fight SARS-Cov-2 infection, its application might be limited due to possible cardiovascular toxicity effects, and it is why other novel treatments involving mesenchymal stem cells (MSCs) are being explored [114].

Lung biopsies of COVID-19 patients show inflammatory infiltrates dominated by lymphocytes and high secretion levels of proinflammatory cytokines, so it is reasonable to recommend the exploration of cytokine antagonists to reduce the severity of the infection, pointing out MSCs due to their ability to inhibit a large number of inflammatory factors that decrease endothelial cell function and cause a respiratory failure resulting in immunomodulated damage to the lungs and other organs, which in turn can lead to ARDS and multiple dysfunctions in other organs [115–118]. Basically, MSC therapeutic effect is through immunomodulation avoiding lung damage caused by inflammation.

To date, around 46 clinical trials with MSCs to treat patients with COVID-19 have been registered at http://www.Clinicaltrials.gov; trials are led by North America, East Asia, and Europe (10, 9, and 8, respectively), and there are 16 trials registered at the Chinese Clinical Trial Registry at http://www.chictr.org.cn adding a total of 62 registered clinical trials of MSC treatment for COVID-19 patients. The first successful treatment of severe COVID-19 patients using MSC determined that intravenous MSC transplantation improved the condition of seven patients with COVID-19 pneumonia (one of them critically ill) that did not respond to conventional treatments. The lung function and symptoms of the seven patients improved significantly 2 to 3 days after the MSC transplant. Also, the severely critically ill COVID-19 patient's breathing difficulty recovered significantly on the third day [119].

## 6. Current Clinical Trials Involving MSC Application and Its Role in Regenerative Medicine

Nowadays, we can consider MSCs as the “novel heart” of regenerative medicine (RM), as RM definition comprises the treatment of diseases and injuries based on healing, tissue regeneration, and restoration of organ function; added to this, the term of healing considers it as the incorporation of the new tissue (product of regeneration) to the “old” and damaged one of the host [120]. If we think of these concepts and recap the capabilities and properties of MSCs, both “stemness” and paracrine activity, it is very clear why MSCs are a promising tool to treat a plethora of conditions in the ambit of RM.

**Figure 3:**
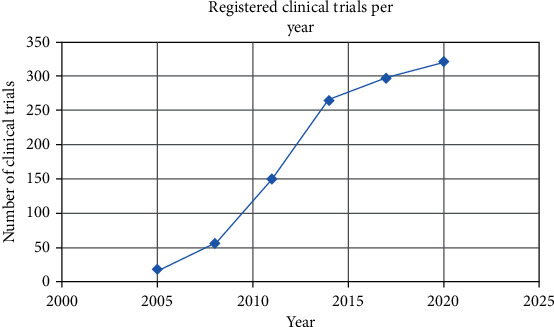
Variations of the number of registered clinical trials through the years at Clinicaltrials.gov.

In agreement with Ballini and colleagues [121] and the previous information, MSCs do show promising outcomes on translational medicine application; however, there are issues to overcome to further escalate these emergent tools. For instance, despite having a clear definition by the ISCT, this only includes MSC potency and phenotypic characteristics, but paracrine activity might vary depending on the donor, culture conditions, source of MSC, and niche among others; added to this, there is also another important matter to consider, the unpredictability of *in vivo* MSC behavior after application, despite the numerous *in vitro* and *in vivo* assays that seek to predict MSC in vivo potency, secretome profiles, and the interaction with their microenvironment. A better understanding and surpassing of these issues might be possible sooner than we think as there is a gradual and constant increase in research and trials related to MSC application, as seen in Figure 3, where it is clear that the registry of trials related to MSCs keeps increasing through the years since the first MSC-related registry in November 1995, which was aimed at treating osteogenesis imperfecta with bone marrow cell transplantation.

**Figure 4:**
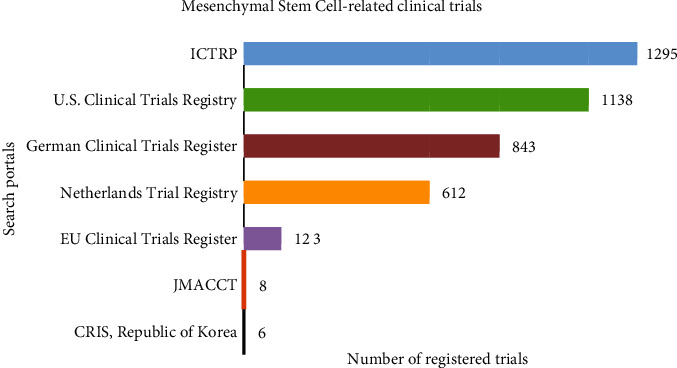
Mesenchymal stem cell-related clinical trial registry among different search portals. Search portals: ICTRP: International Clinical Trials Registry Platform (https://apps.who.int/trialsearch/AdvSearch.aspx); Chinese Clinical Trial Registry (http://www.chictr.org.cn/index.aspx); U.S. Clinical Trials Registry (http://www.clinicaltrials.gov/); German Clinical Trials Register (https://www.drks.de/drks_web/setLocale_EN.do); Netherlands Trial Registry (https://www.trialregister.nl/trials); EU Clinical Trials Register (https://www.clinicaltrialsregister.eu/); JMACCT: Center for Clinical Trials, Japan Medical Association (http://www.jmacct.med.or.jp/); CRIS: Clinical Research Information Service Republic of Korea (https://cris.nih.go.kr/cris/en/).

Moreover, advances in MSCs and translational medicine are reflected directly in the number of registered clinical trials around the world; numbers keep increasing day by day, and as you read this review, there might be new recently added trials. These registries and numbers can be consulted using several search portals (Figure 4), where the highest number of registered trials corresponds to the International Clinical Trials Registry Platform (ICTRP) which may also comprise some of the trials registered on the other databases; also, it is noted that the U.S. Clinical Trials Registry remains as one of the most relevant platforms to search and register clinical trials related to MSCs having a total of 1,138 registries.

**Figure 5:**
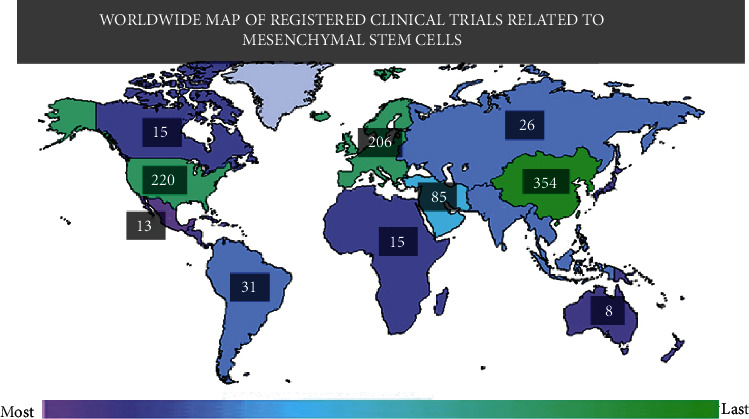
Worldwide map of registered clinical trials employing mesenchymal stem cells. The color indicates the number of studies within a region; exact study numbers are enclosed into labels. The number of studies by region: Africa 15, Central America 10, East Asia 354, Japan 9, Europe 206, Middle East 85, North America 230, Canada 15, Mexico 3, United States 220, North Asia 26, Pacifica 8, South America 31, South Asia 29, and Southeast Asia 29. Extracted and modified from http://www.clinicaltrials.gov.

Furthermore, the distribution of registered clinical trials worldwide according to http://www.clinicaltrials.gov is described in Figure 5 and shows that East Asia, North America, and Europe are the main locations where most clinical research is done on the therapeutic effects of MSCs; these three regions together add 50% of the total registered clinical trials around the world, and consistent with the data presented by Wei et al. in 2013 [4], it is clear that the MSC trend in the clinical trial registry keeps growing especially in Asia and North America.

**Figure 6:**
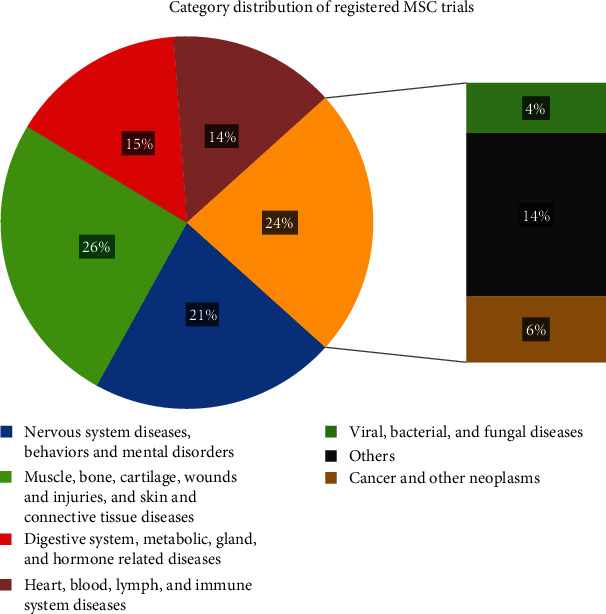
Category distribution and percentage of registered clinical trials.

Also, registered trials are classified according to the targeted disease or condition (Figure 6); the highest percentages correspond to trials involving muscle, bone, cartilage, wounds, injuries, skin, and connective tissue diseases (26%); nervous system diseases and behavior and mental disorders (21%); and others (14%) including eye, mouth, tooth, urinary tract, sexual organ, and general pathologies; all these main categories are often subdivided into more specific categories.

Consistent with the data presented by [4], it is clear that the MSC trend in the clinical trial registry keeps growing especially in Asia and North America.

## 7. Conclusions

Among all the other stem cell types, MSCs remain the most relevant stem cell technology used in a plethora of diseases due to their immunomodulatory capacity and their differentiation potential. However, the complete understanding of their interactions in the in vivo models remains a challenge, especially because of some contradictory reports on MSC activity, as MSC cancer treatment has shown both protumorigenic and antitumor effects. On the other hand, the administration of secretome products via EVs represents a window of opportunity to avoid employing whole cells; this needs further studies but may guarantee the security of MSC therapies. Finally, MSCs represent a novel tool for the development of new therapies, and it will not be a surprise if further MSC-based therapies get FDA approval.

## Figures and Tables

**Table 1:** Sources of mesenchymal stem cells and their differentiation potential.

Source	Differentiation potential (cell lineage)	Reference
Bone marrow	(i) Hepatocytes (ii) Pancreatic cells (iii) Neuron-like cells (iv) Cardiomyocytes	[19–22]
Adipose tissue	(i) Neuron-like cells (ii) Cardiomyocytes (iii) Hepatocytes (iv) Pancreatic cells	[22–25]
Dental pulp and other dental tissues	(i) Pancreatic *β* cell-like cells (ii) Hepatocytes (iii) Myocytes (iv) Pancreatic cells (v) Neuron-like cells	[26–30]
Umbilical cord blood (UCB) and UC	(i) Hepatocytes (ii) Pancreatic islet-like cells (iii) Neurons	[31–33]
Wharton's jelly	(i) Hepatocytes (ii) Neurons	[34, 35]
Dermal tissue-derived MSCs	(i) Myocytes	[36]

**Table 2:** Some of the molecules secreted by MSCs and their functions.

Product of secretome		Role	Ref.
Extracellular vesicles		(i) Participation in intercellular communication (ii) Carrying of bioactive molecules (GFs, proteins, lipids, miRNAs, tRNA, and lncRNA); this might be involved in angiogenic modulation	[45, 46]
Cytokines	IL-6	(i) Control of macrophage T activation (ii) Proinflammatory inflammatory acute phase response induced by tissue damage	[47, 48]
	IL-10	(i) Promotion of wound and tissue repair (ii) Immunosuppression, avoiding of autoimmune injuries (iii) Modulation of macrophage and neutrophil functions	[49, 50]
	IL-1Ra	(i) Suppression of Th17 differentiation (ii) Inhibition of B cell differentiation (iii) Control of macrophage polarization towards M2 phenotype	[51, 52]
	PGE2	(i) Facilitates MSC migration (ii) Anti-inflammatory	[53, 54]
	LIF	(i) Modulation of lymphocyte generation (ii) Immunosuppression	[55]
Growth factors	VEGF	(i) Angiogenesis (ii) Differentiation towards endothelial cells (iii) Protection against hyperoxia tissue injuries	[56, 57]
	TGF-*β*	(i) M2 macrophage polarization (ii) Inhibition of human T cells	[58, 59]
	HGF-1	(i) Angiogenesis improvement	[60]
Other molecules	Gal-9	(i) Suppression of Th1 and Th17 cell differentiation (ii) Antiproliferative effects on T cells	[61, 62]
	Gal-1	(i) Antiproliferative effects on CD4^+^ and CD8^+^ T cells (ii) Modulation of cytokine release (iii) Modulation of T cell response	[63, 64]
	TSG-6	(i) Downregulation of TLR2/NF-*κ*B signaling (ii) A decrease in the expression of inflammatory cytokines	[65]
	MpCCL2	(i) Regulation of Th17 CD4 T cell activation	[66]

## Data Availability

The data supporting this systematic review are from previously reported studies and datasets from clinical trials registries, which have been cited. The processed data are available from the corresponding author upon request.
